# Supplementary material for: Bna.EPF2 Enhances Drought Tolerance by Regulating Stomatal Development and Stomatal Size in Brassica napus
Source: Int J Mol Sci. 2023 Apr 28;24(9):8007. doi: 10.3390/ijms24098007 (PMC10179174; doi:10.3390/ijms24098007)
Supplement: Supplementary file 1 [file ijms-24-08007-s001.zip › ijms-2292434-supplementary.pdf]

Bna.EPF2 Enhances Drought Tolerance by Regulating Stomatal Development and stomatal size in *Brassica napus*

Peipei Jiao[1,2], Shaoping Chen[1], Jiali Li[1], Yuanlin Liang[1], Honghong Hu[1*]

Supplemental Files

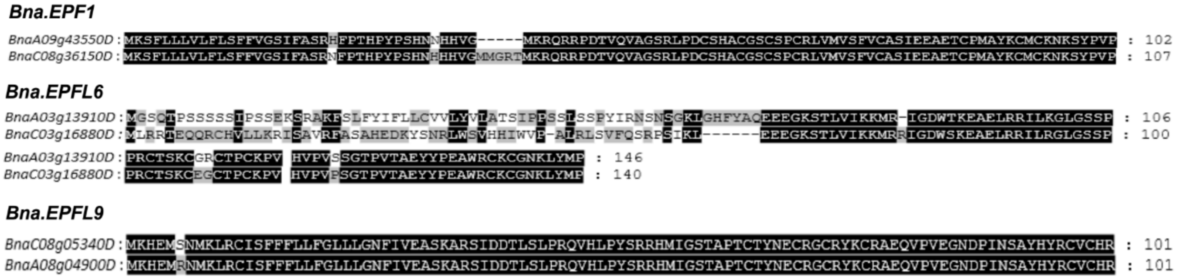

Supplemental Figure S1. The sequence similarity of *EPF1*, *EPFL6*, and *EPFL9* homologs in *Brassica napus*. Alignment was performed with ClustalX (version 1.83) in multiple alignment mode and edited with GeneDoc1.0 software (www.psc.edu/biomed/genedoc). Black shaded indicated high sequence similarity, light gray indicated sequence similarity was low.

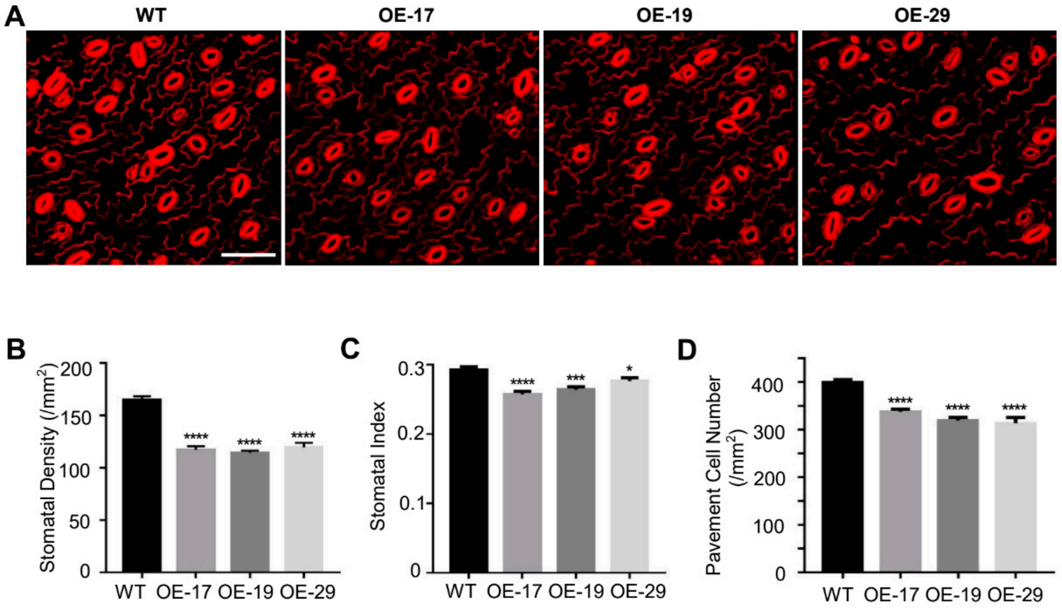

Supplemental Figure S2. The stomatal density in cotyledons of *BnA.EPF2* overexpression was lower compared to WT. (A) Imaging of epidermis of 15 day-after-germination cotyledons of WT and *Bna.EPF2* overexpression lines. Cotyledon epidermal layers were stained by propidium iodide (PI) for 5 min and the images were taken by a confocal microscopy. Bar = 100 µm. (**B**-**D**) Statistical analyses of stomatal density (**B**), stomata index (**C**) and pavement cell density (**D**) in 15 day-after-germination cotyledons of WT and *Bna.EPF2* overexpression lines. Values are means ± SE (each with at least 20 leaves). *, $P < 0.05$; ***, $P < 0.001$; ****, $P < 0.0001$; Student's t-test.

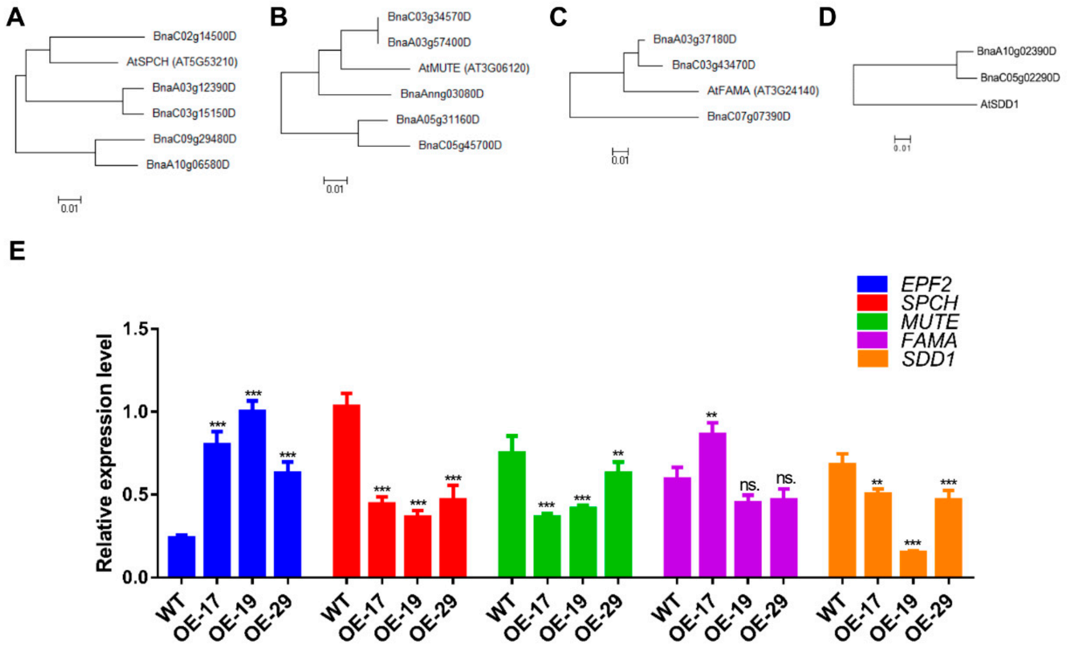

Supplemental Figure S3. Expression levels of homologous genes involved in stomatal development pathway in *Bna.EPF2* overexpression lines. (A-D) The phylogenetic tree of the homologous genes from *Arabidopsis thaliana* and *Brassica napus* were constructed using the MEGA6.06 software. **(E)** Gene expression levels of these homologs in WT, and *Bna.EPF2* overexpression lines. Values are means ± SE, three biological replicates; No significantly difference (ns.), P ≥ 0.05; **, $P < 0.01$; ***, P <0.001; Student's t-test.

Table S1. **Primers used in this research**

Primer name	Primer sequence（5’-3’）
NPTII-F	GGAAAACGATTCCGAAGCCCAA
NPTII-R	ACAACAGACAATCGGCTGCTCT
epf2-L	TAAAACCTCTGCCTCAACCAG
epf2-R	TTACCGGTATGATGGAGATGG
LBb l.3	ATTTTGCCGATTTCGGAAC
Bna.EPF2-F	TGCTCTAGAATGAAGAAGTTTCTCCGCACG
Bna.EPF2-R	TCCCCCGGGTCAAGCCCTAGATGGCACGTG
Bna.EPF2-pF	GCCCAAGCTTCCTAATCTGAATTACGTTTAAGCAA
Bna.EPF2-pR	GCGCGGATCCGTTTTATTTTATTTTTGTCTCAACAAG
35S::JW771-F	ACGTTCCAACCACGTCTTC
Bna.EPF2-qL	CCCACCACAAGGAAATAAAG
Bna.EPF2-qR	GGAACAATCAGGTAAACTCG
Bna.SDD1-qL	GGAAGCAGAGGAGAGAAGGTG
Bna.SDD1-qR	ACTCCTAACTCGCTGCATCAC
Bna.FAMA-qL	GACTATCGCTGCTTTGGAGG
Bna.FAMA-qR	CACGTGTTTATACTTACTTGCCTTG
Bna.MUTE-qL	TCTCGAGTCCAAGAAACGCC
Bna.MUTE-qR	GTGGCTGCGGCTAATATGGA
Bna.SPCH-qL	CCCTCGTCCTTCACCAAATGT
Bna.SPCH-qR	GAGGGGTTCGAGGGCTTATG
RT-*Bna.*actin-F	CCCTGGAATTGCTGACCGTA
RT-*Bna.*actin-R	TGGAAAGTGCTGAGGGATGC
